# CT-based radiomics signature of visceral adipose tissue and bowel lesions for identifying patients with Crohn’s disease resistant to infliximab

**DOI:** 10.1186/s13244-023-01581-9

**Published:** 2024-01-30

**Authors:** Yangdi Wang, Zixin Luo, Zhengran Zhou, Yingkui Zhong, Ruonan Zhang, Xiaodi Shen, Lili Huang, Weitao He, Jinjiang Lin, Jiayu Fang, Qiapeng Huang, Haipeng Wang, Zhuya Zhang, Ren Mao, Shi-Ting Feng, Xuehua Li, Bingsheng Huang, Zhoulei Li, Jian Zhang, Zhihui Chen

**Affiliations:** 1 Department of Radiology, The First Affiliated Hospital, Sun Yat-Sen University, 58 Zhongshan II Road, Guangzhou, Guangdong 510080 People’s Republic of China; 2 Medical AI Lab, School of Biomedical Engineering, Shenzhen University Medical School, Shenzhen University, Shenzhen, Guangdong People’s Republic of China; 3 Zhongshan School of Medicine, Sun Yat-Sen University, 74 Zhongshan II Road, Guangzhou, Guangdong People’s Republic of China; 4 Department of Gastroenterology, The Sixth Affiliated Hospital, Sun Yat-Sen University, Yuancun Er Heng Road, No. 26, Guangzhou, Guangdong People’s Republic of China; 5 Department of Gastrointestinal Surgery, The First Affiliated Hospital, Sun Yat-Sen University, 58 Zhongshan II Road, Guangzhou, Guangdong People’s Republic of China; 6 Department of Gastroenterology, The First Affiliated Hospital, Sun Yat-Sen University, 58 Zhongshan II Road, Guangzhou, Guangdong People’s Republic of China; 7 Shenzhen-Hong Kong Institute of Brain Science-Shenzhen Fundamental Research Institutions, Shenzhen, Guangdong People’s Republic of China; 8 Health Science Center, School of Biomedical Engineering, Shenzhen University, Shenzhen, Guangdong People’s Republic of China; 9 Guangxi Hospital Division of The First Affiliated Hospital, Sun Yat-sen University, Nanning, Guangxi People’s Republic of China

**Keywords:** Computed tomography enterography, Crohn’s disease, Infliximab therapy, Primary nonresponse, Radiomics

## Abstract

**Purpose:**

To develop a CT-based radiomics model combining with VAT and bowel features to improve the predictive efficacy of IFX therapy on the basis of bowel model.

**Methods:**

This retrospective study included 231 CD patients (training cohort, *n* = 112; internal validation cohort, *n* = 48; external validation cohort, *n* = 71) from two tertiary centers. Machine-learning VAT model and bowel model were developed separately to identify CD patients with primary nonresponse to IFX. A comprehensive model incorporating VAT and bowel radiomics features was further established to verify whether CT features extracted from VAT would improve the predictive efficacy of bowel model. Area under the curve (AUC) and decision curve analysis were used to compare the prediction performance. Clinical utility was assessed by integrated differentiation improvement (IDI).

**Results:**

VAT model and bowel model exhibited comparable performance for identifying patients with primary nonresponse in both internal (AUC: VAT model vs bowel model, 0.737 (95% CI, 0.590–0.854) vs. 0.832 (95% CI, 0.750–0.896)) and external validation cohort [AUC: VAT model vs. bowel model, 0.714 (95% CI, 0.595–0.815) vs. 0.799 (95% CI, 0.687–0.885)), exhibiting a relatively good net benefit. The comprehensive model incorporating VAT into bowel model yielded a satisfactory predictive efficacy in both internal (AUC, 0.840 (95% CI, 0.706–0.930)) and external validation cohort (AUC, 0.833 (95% CI, 0.726–0.911)), significantly better than bowel alone (IDI = 4.2% and 3.7% in internal and external validation cohorts, both *p* < 0.05).

**Conclusion:**

VAT has an effect on IFX treatment response. It improves the performance for identification of CD patients at high risk of primary nonresponse to IFX therapy with selected features from RM.

**Critical relevance statement:**

Our radiomics model (RM) for VAT-bowel analysis captured the pathophysiological changes occurring in VAT and whole bowel lesion, which could help to identify CD patients who would not response to infliximab at the beginning of therapy.

**Key points:**

• Radiomics signatures with VAT and bowel alone or in combination predicting infliximab efficacy.

• VAT features contribute to the prediction of IFX treatment efficacy.

• Comprehensive model improved the performance compared with the bowel model alone.

**Graphical abstract:**

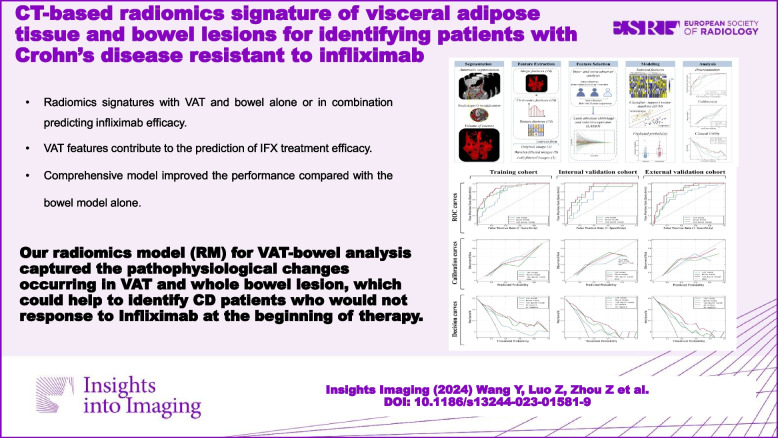

**Supplementary Information:**

The online version contains supplementary material available at 10.1186/s13244-023-01581-9.

## Introduction

Crohn’s disease (CD) is a subtype of inflammatory bowel disease (IBD), which results in progressive intestinal damage and disability [1]. Infliximab (IFX), a monoclonal antibody selectively targeting tumor necrosis factor-α (TNF-α), offers mainstay for managing CD patients with moderate to severe inflammation and improves mucosal healing and clinical remission [2–4]. However, approximately 13–40% of CD patients resist to the initial IFX therapy (primary nonresponse, PNR) [5, 6], related to elevated cost and even risk of severe side effects, and require the implementation of “precision medicine” [7, 8]. Thus, it is necessary to screen patients sensitive to IFX therapy in advance and develop prognostic tools for outcomes.

Although several risk factors have been identified to be associate with therapeutic nonresponse [2, 5, 7], conflicting results or lack of validation still obstruct the strategy on improving therapeutic outcome, due to the unclear mechanisms of PNR. Recently, visceral adipose tissue (VAT), involved in the pathogenesis of CD and associated with more complex disease phenotype [9], has shown strong association with recurrence and postoperative complications [10, 11]. Besides, evidence indicates that VAT as source of proinflammatory substances associates with chronic intestinal inflammation [12, 13]. Therefore, VAT might be a useful predictor for IFX response.

Computed tomography (CT) enables noninvasively measurement of VAT [14]. Previous studies have quantitative analyzed metrics of VAT volume to clarify the relationship between adipose and therapeutic response [15]. Furthermore, radiomics can efficiently extract numerous imaging features imperceptible to the naked eyes [16] and allow more accurate identification of the features of bowel lesions in CD patients [17]. Therefore, the use of CT-based radiomics to extract effective features from both intestinal lesions and VAT may potentially enhanced pharmacotherapy response prediction.

Given the prior highlighted values of radiomics and the non-negligible role of VAT in disease progression, we aim to develop a comprehensive radiomics model (VAT-bowel model) based on the pretreatment CT features of VAT and bowel features to compare with bowel model alone and to explore whether VAT can further improve the predictive efficacy on the basis of bowel model.

## Methods

### Patient and study design

In this retrospective study, a total of 231 patients with CD who underwent computed tomography enterography (CTE) before standardized IFX treatment for clinically and/or endoscopically active disease were consecutively recruited between January 2013 and December 2020 in two tertiary IBD centers under the institutional ethics review from both the Sixth Affiliated Hospital of Sun Yat-Sen University (center 1) and the First Affiliated Hospital of Sun Yat-Sen University (center 2).

The inclusion criteria were as follows: (a) patients underwent CTE within 1 month prior to IFX therapy; (b) treated with regularly standardized IFX induction therapy (5 mg/kg at weeks 0–2 to 2–6 induction, week 14 evaluation; (c) the absence of previous anti-TNF therapy; and (d) performed simple endoscopic score for Crohn’s disease (SES-CD) > 3 of standard endoscopy within 0.5 months prior to IFX therapy. The exclusion criteria were as follows: (a) poor CTE image quality that hindered analysis; (b) history of enterotomy, which may influence the nature radiomic features; (c) lack of posttreatment endoscopic comparison; and (d) poorly defined intestinal wall and VAT due to severe effusion around the lesion.

According to the inclusion and exclusion criteria, 231 patients with CD were included (Supplement Figure 1). Patients with CD who are at the First Affiliated Hospital of Sun Yat-Sen University between January 2013 and December 2020 were semirandomly allocated to training cohort and test cohort 1, maintaining a ratio of 7:3 (112 patients:48 patients). Another 71 CD patients who underwent IFX treatment in the Sixth Affiliated Hospital of Sun Yat-Sen University between January 2018 and December 2020 were allocated as test cohort 2.

### Definition of primary response and nonresponse to infliximab therapy

Patients with CD in the two centers received standard IFX induction at the weeks 0–2 to 2–6, with a dosage of 5 mg/kg. At week 14, clinical symptoms, endoscopy, laboratory examination, and anti-IFX drug levels were collected from each patient to assess the efficacy of IFX therapy. Additionally, information was collected on whether IFX was used as monotherapy or in combination with an immunomodulator. Patients who did not show satisfactory improvement in a global physician assessment and required treatment changes such as dose escalation, corticosteroid addition, agent switch, or surgery were defined as having PNR. Otherwise, they were classified as primary response to IFX (PR). Besides, patients categorized as PR also needed to conform to the decrease of 50% in SES-CD relative to the baseline as recommended in expert consensus and prior reported clinical trials [18, 19]. Two gastroenterologists (C. Z. and H. Q.) retrospectively evaluated the SES-CD through the report description and endoscopic pictures.

### Development and validation of VAT radiomics model

**Fig. 1:**
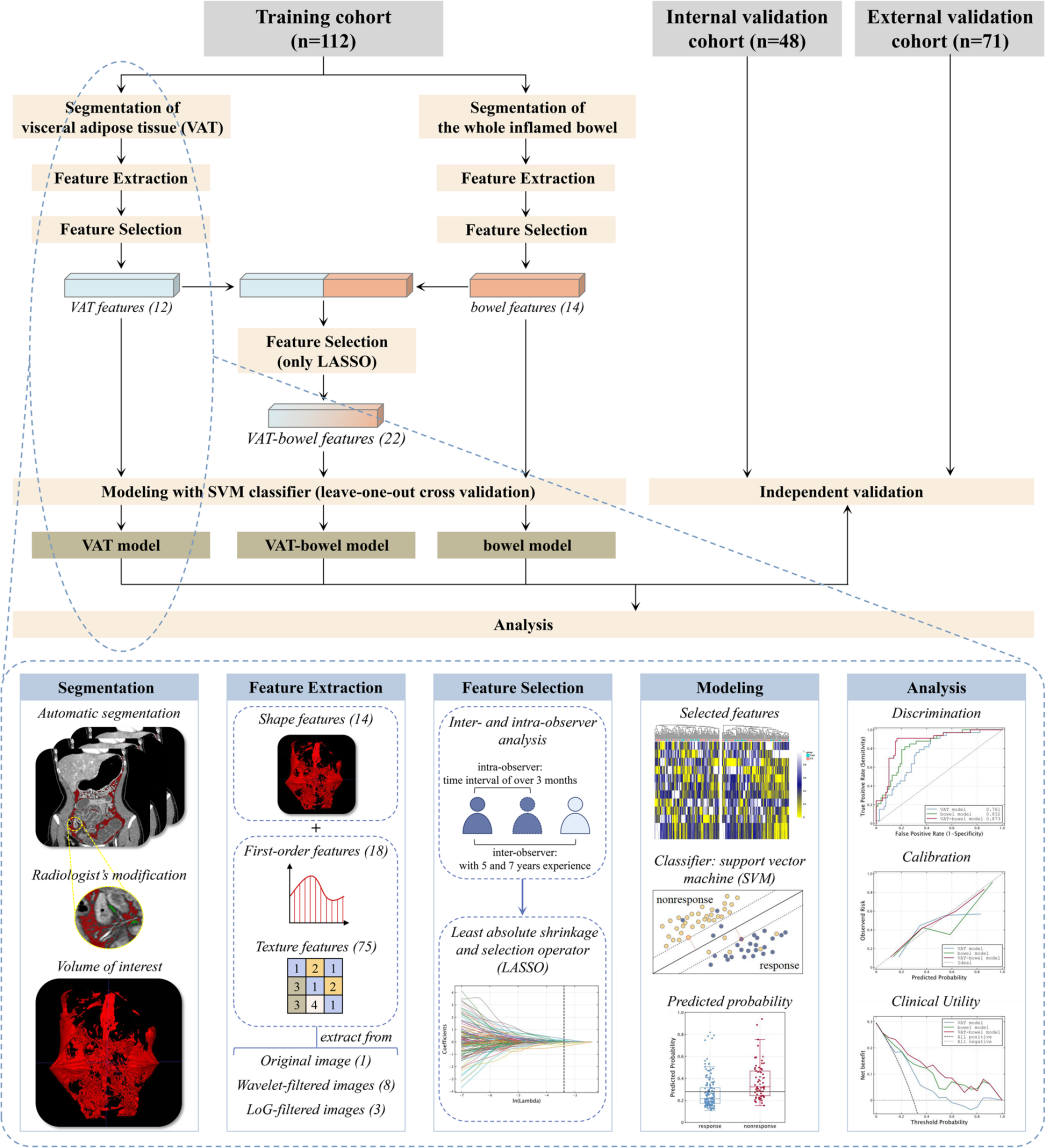
The study flow chart (upper) and the radiomics analysis workflow (lower) (VAT model, radiomics model based on features extracted from visceral adipose tissue; bowel model, radiomics model based on features extracted from the inflamed bowel; VAT-bowel model, a combination of the VAT model and bowel model)

The study flowchart and the radiomics analysis workflow are shown in Fig. 1, which illustrates the procedure for the development of the VAT model, bowel model and VAT-bowel model, and details of the development.

### Radiomics features extraction and selection

**Fig. 2:**
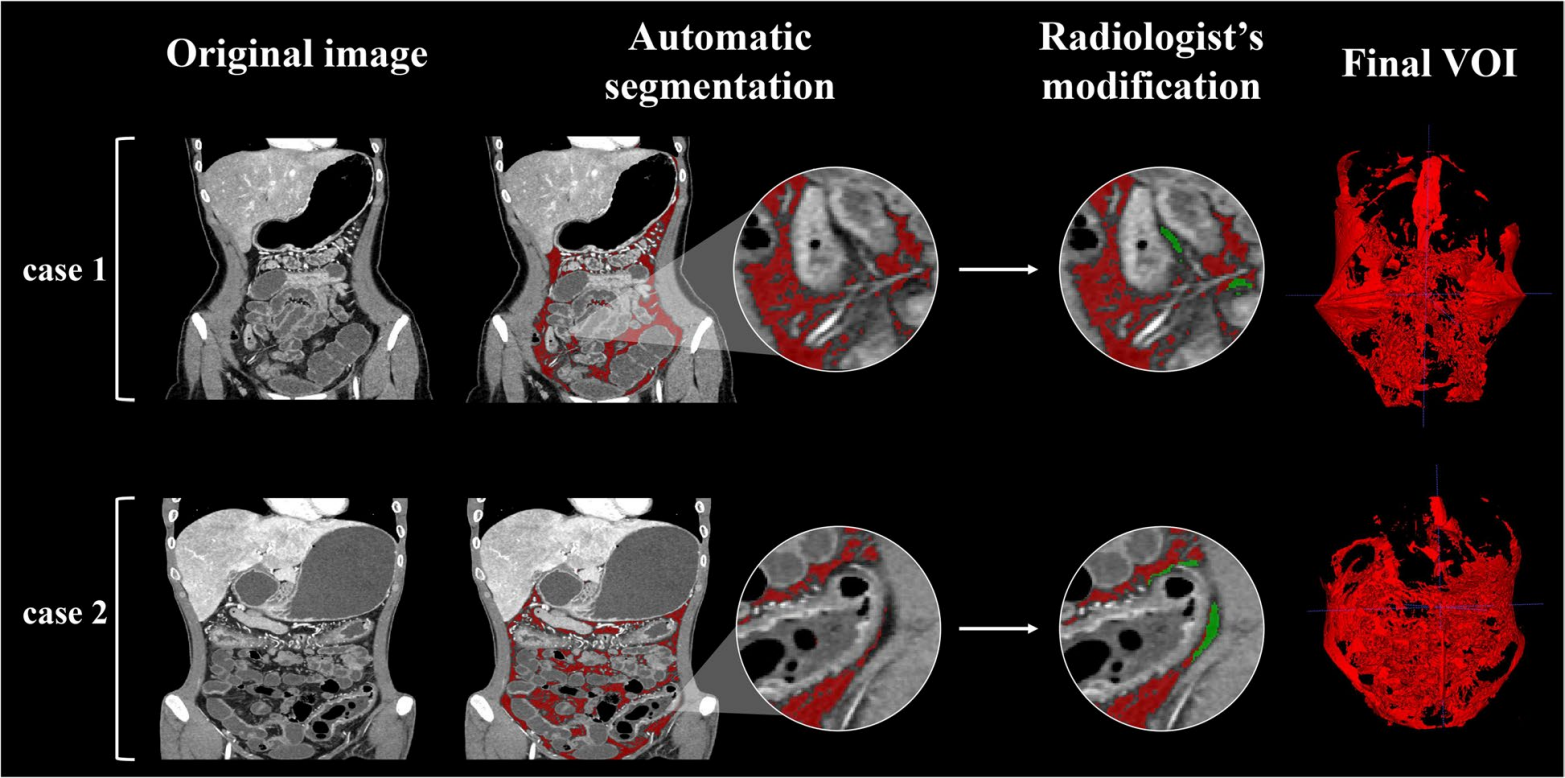
The process of VOI segmentation on two cases, including automatic segmentation and radiologist’s modification. The dice similarity coefficients for cases 1 and 2 are 0.971 and 0.957, respectively. The red regions are the automatic segmentation results generated by the nnU-net, while the green regions indicate the area, modified from radiologists (VOI, volume of interest)

Given the extensive and intricate distribution of VAT, it was automatically segmented on CT images by utilizing a deep learning-based framework called nnU-Net [20], which has been widely used in medical image segmentation task. To do so, two radiologists (Z.R. and Y.W.) scrutinized and modified the segmentation results with open-source software ITK-SNAP (version 3.4.0; https://www.itksnap.org) to determine the final volume of interest (VOI) in this study. The process of VOI segmentation on two cases is shown in Fig. 2, and the details are described in Supplementary materials A1.

From each VOI on CT images, a total of 1130 radiomics features were extracted with the following categories: shape features, first-order features, texture features, and these imaging features transformed by different filters. The feature extraction was performed in the Python environment (version 3.6; https://www.python.org/) using PyRadiomics toolkit (version 3.0.1; https://pyradiomics.readthedocs.io/en/latest/index.html). More details are provided in Supplementary materials A2.

The reproducibility of the extracted radiomics features was assessed through inter- and intra-observer analysis with calculation of intraclass correlation coefficients (ICCs). Subsequently, a refinement process was conducted to purify the radiomics features (Supplementary materials A3).

### Radiomics model development and validation

Based on the selected features, a binary classification model was built for distinguishing between PR and PNR by using a support vector machine (SVM) classifier and detailed in Supplementary materials A4.

The validation of the VAT radiomics model was conducted on the internal and external validation cohorts using the optimal subset of features and model parameters obtained from the training cohort. The final prediction probability for each sample in the validation cohorts was derived by averaging the outputs of all models generated by the LOOCV strategy during the training phase. The development and validation of the radiomics model were performed in Python environment (version 3.6; https://www.python.org/) using scikit-learn toolkit (version 19.0; https://scikit-learn.org/stable).

### Development and validation of bowel and VAT-bowel radiomics models

In our previous study [17], a radiomics model based on the entire inflamed bowel in CTE images (bowel model) was established for identification of PNR to IFX therapy. To further improve its performance, radiomics features of VAT would be added to the bowel model to construct a comprehensive (VAT-bowel) radiomics model. The constructing process of both bowel and VAT-bowel radiomics model is referred to Fig. 1.

For the development of bowel model, the extraction and selection of bowel features were consistent with our previous study. For the development of VAT-bowel model, we combined the selected VAT features and bowel features and further selected them by using LASSO to obtain the optimal subset of VAT-bowel features. Both models were built by utilizing the SVM classifier and the LOOCV strategy, following the same development and validation process as the VAT model.

### Statistical analysis

#### Evaluation of sample size

The predictive performance of the VAT model was assessed by using receiver operating characteristic (ROC) analysis. The area under the ROC curve (AUC) and the corresponding 95% confidence interval (CI) were calculated.

A sample size of 48 patients (34 PR and 14 PNR) is required for the LOOCV modeling of IFX therapy prediction based on the following conditions by using MedCalc Statistical Software (version 15.8; http://www.medcalc.org/) and detailed in Supplementary material A5.

### Predictive performance comparison of bowel and VAT-bowel radiomics models

In order to explore whether and to what extent the efficacy of the model could be improved when incorporating VAT features into our previously developed bowel model, a comparative analysis was conducted between the performance of the bowel model and that of the VAT-bowel radiomics model using the following methods. DeLong’s test was employed to compare the AUCs of the models when McNemar’s test was used to compare the accuracy. The integrated discriminant improvement (IDI) index was calculated to compare the incremental predictive utility between the two radiomics models. Additionally, the Hosmer-Lemeshow goodness-of-fit test was utilized to evaluate the calibration performance of the models. The clinical utility of each model was assessed by calculating the net benefit at different threshold probabilities and conducting a decision curve analysis.

A two-tailed *p* value less than 0.05 was considered statistically significant. All statistical analyses were performed with R statistical software (version 4.0.4; http://www.r-project.org/).

## Results

### Patient characteristics

During the follow-up period, 33 patients (33/112, 29.5%) from the training cohort and 36 patients (36/119, 28.1%) (14/48 from test cohort 1 and 22/71 from test cohort 2) from the total test cohort experienced IFX treatment. Four univariate analysis recognized clinical factors in PNR, including BMI, CRP, Hb, and ALB, and exhibited significant difference compared to PR in the training cohort (Table 1, *p* < 0.05).

**Table 1:** Characters of patients in the training and test cohorts

	Training cohort (n = 112)			Test cohort 1 (n = 48)			Test cohort 2 (n = 71)		
Characters	Response (n = 79)	PNR (n = 33)	*p* value	Response (n = 34)	PNR (n = 14)	*p* value	Response (n = 49)	PNR (n = 22)	*p* value
Age^a^	25.0 (9–56)	24.2 (12–41)	0.434	25 (14–49)	22 (14-46)	0.220	26 (16–39)	24 (13–50)	0.910
Sex^b^			0.983			0.320			0.999
Male	58 (73.4%)	25 (75.8%)		27 (74%)	8 (57%)		44 (90%)	15 (68%)	
Female	21 (26.6%)	8 (24.2%)		9 (26%)	6 (43%)		5 (10%)	7 (32%)	
BMI (kg/m^2^)^c^	18.3 ± 2.6	17.2 ± 2.6	0.058	18.4 ± 2.9	17.1 ± 2.0	0. 081	20.1 ± 2.9	18.9 ± 3.1	0.087
CRP (mg/L)^c^	26.2 ± 25.8	37.6 ± 30.6	0.065	33.8 ± 28.3	47.9 ± 28.1	0.100	28.6 ± 30.5	35.1 ± 32.0	0.180
ESR (mm/h)^c^	50.4 ± 26.9	57.0 ± 30.4	0.283	57.4 ± 32.4	73.2 ± 35.0	0.140	38.6 ± 25.3	54.7 ± 30.2	0.039*
Hb (g/L)^c^	111.8 ± 17.8	102.1 ± 17.5	0.004*	107.3 ± 24.7	105.6 ± 12.4	0.360	99.4 ± 14.8	140.3± 17.3	0.140
ALB (g/L)^c^	34.8 ± 4.9	30.8 ± 4.9	< 0.001*	33.3 ± 6.1	33.7 ± 4.3	0.950	39.5 ± 9.2	35.6 ± 5.3	0.032*
PLT (10^3^/mm^3^)^a^	357 (137–604)	407 (283–491)	0.057	444 (147–808)	376 (115–763)	0.092	355 (145–701)	408 (226–742)	0.190
Disease behavior^b^									
B1 (inflammatory)^d^	45 (57.0%)	13 (39.4%)		18 (53%)	7 (50%)	0.691	40 (82%)	17 (77%)	0.865
B2 (stricturing)^d^	25 (31.6%)	15 (45.5%)		12 (35%)	5 (36%)	0.794	1 (2.0%)	1 (4.5%)	0.999
B3 (penetrating)^d^	9 (11.4%)	5 (15.1%)		4 (12%)	2 (14%)	0.999	8 (16%)	4 (18%)	0.999
Perianal disease^d^	9 (11.4%)	6 (18.2%)		4 (12%)	2 (14%)	0.990	31 (63%)	11 (50%)	0.290
Disease location^b^			0.362			0.347			0.760
L1 (ileal disease)	13 (16.5%)	2 (6.1%)		4 (12%)	3 (21%)		8 (16%)	2 (9.1%)	
L2 (colonic disease)	6 (7.6%)	3 (9.1%)		0 (0%)	2 (14%)		5 (10%)	3 (14%)	
L3 (ileocolonic disease)	60 (75.9%)	28 (84.8%)		30 (88%)	9 (64%)		36 (73%)	17 (77%)	
Duration of disease^b^			0.495			0.754			0.360
(< 2 years)	50 (63.3%)	16 (48.5%)		23 (68%)	10 (71%)		24 (49%)	9 (41%)	
(2–5 years)	14 (17.7%)	8 (24.2%)		10 (29%)	3 (21%)		12 (24%)	9 (41%)	
(> 5 years)	15 (19.0%)	9 (27.3%)		1 (2.9%)	1 (7.1%)		13 (27%)	4 (18%)	
Medications^b^									
Immunomodulator	18 (22.8%)	13 (39.4%)	0.119	5 (15%)	2 (14%)	0.679	25 (51%)	10 (45%)	0.660
Corticosteroids	10 (12.7%)	5 (15.2%)	0.764	5 (15%)	2 (14%)	0.999	12 (24%)	4 (18%)	0.347
Aminosalicylates (5‐ASAs)	31 (39.2%)	20 (60.6%)	0.063	13 (38%)	2 (14%)	0.177	29 (59%)	11 (50%)	0.298
Combination immunosuppression	34 (43.0%)	14 (42.4%)	0.401	16 (47%)	4 (29%)	0.361	21 (43%)	6 (27%)	0.210
CDAI^c^	219 ± 81.5	246 ± 65.9	-	228 ± 64	265 ± 95	-	251 ± 72	273 ± 66	-
SES‐CD^a^	16.1 (4–41)	17.2 (0–41)	-	17 (3–31)	13 (0–28)	-	17 (7–30)	20 (9–34)	-

*Abbreviations*: *ALB* albumin, *BMI* body mass index, *CDAI* Crohn's Disease Activity Index, *CRP* C-reactive protein, *ESR* erythrocyte sedimentation rate, *Hb* Hemoglobin, *PLT* platelets, *PNR* primary nonresponse, *SES‐CD* Simple Endoscopic Score for Crohn Disease

^a^Nonnormally distributed continuous variables, expressed as median (interquartile range), line Mann–Whitney U test

^b^Categorical variables, expressed as frequencies (proportions), line χ2 test

^c^Normally distributed continuous variables, expressed as mean ± standard deviation, line independent t test

^d^The sum of the subgroups does not equal with the total cohort, due to overlapping of B1 and B2 categories. The p value was calculated based on each subgroup

*p value was derived from the univariable association analyses between each characteristic and response/nonresponse status

### Development and validation of VAT radiomics model

#### Radiomics features selection and predictive performance validation for the radiomics model

**Fig. 3:**
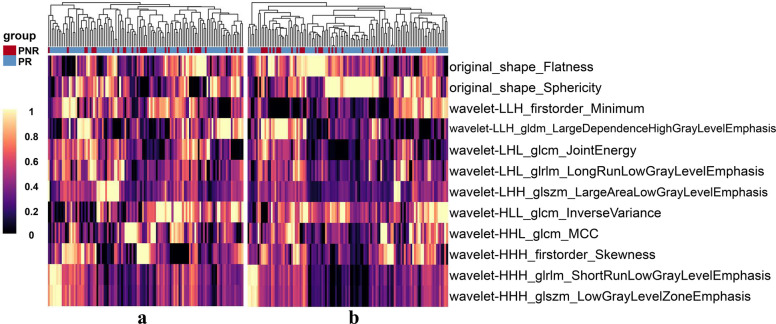
Heatmaps generated by unsupervised hierarchical clustering of the 12 selected features of the VAT model in the (**a**) training cohort and (**b**) total validation cohort, respectively. The feature values are standardized to the range of [0, 1] in order to achieve a clear view. Each row of the heatmap is one selected radiomics feature, and each column is a sample (PNR, red; PR, blue). At the top, generated dendrogram represents samples with similar information determined by clustering (PNR, primary nonresponse; PR, primary response to infliximab therapy; VAT model, radiomics model based on features extracted from visceral adipose tissue)

Based on LASSO algorithm, 12 features were selected for training or validation cohorts (Supplementary materials A6, Fig. 3). The correlation coefficients were shown in Table 2.

**Table 2:** The selected radiomics features of the VAT radiomics model and the corresponding coefficients

Radiomics feature	Coefficient (absolute value)
original_shape_Flatness	0.067293
original_shape_Sphericity	0.047925
wavelet-LHL_glrlm_LongRunLowGrayLevelEmphasis	0.035222
wavelet-HHH_firstorder_Skewness	0.034753
wavelet-HHH_glszm_LowGrayLevelZoneEmphasis	0.032765
wavelet-HLL_glcm_InverseVariance	0.019269
wavelet-HHL_glcm_MCC	0.011879
wavelet-LHL_glcm_JointEnergy	0.008703
wavelet-LLH_firstorder_Minimum	0.005895
wavelet-LHH_glszm_LargeAreaLowGrayLevelEmphasis	0.005520
wavelet-LLH_gldm_LargeDependenceHighGrayLevelEmphasis	0.005261
wavelet-HHH_glrlm_ShortRunLowGrayLevelEmphasis	0.004115

The VAT radiomics model was the model developed based on radiomics features extracted from visceral adipose tissue (VAT). The coefficient of each radiomics feature was generated by the least absolute shrinkage and selection operator algorithm and presented as absolute value. Each feature was named by concatenating the image type from which the feature was extracted, feature group and feature name by underline. For example, original_shape_Flatness was a feature extracted from the original image, shape group, and the feature name was *Flatness*. Glrlm, gray-level run length matrix; glszm, gray-level size zone matrix; glcm, gray-level co-occurrence matrix; gldm, gray-level dependence matrix; all features above belong to texture features

**Fig. 4:**
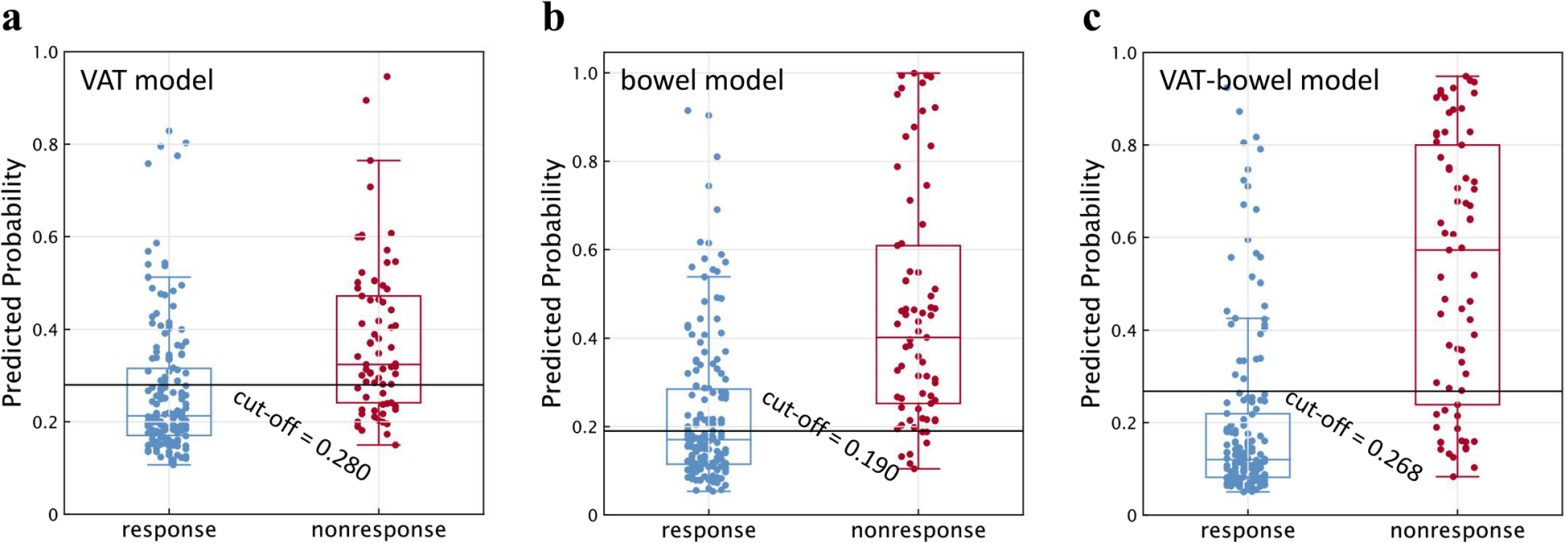
Scatter plots of the predicted probabilities of the (**a**) VAT model, (**b**) bowel model, and (**c**) VAT-bowel model for distinguishing PR from PNR on all data cohorts. A horizontal solid line is drawn at each plot map and indicates the optimal cut-off value of 0.280, 0.190, and 0.268, respectively. The points above the solid line are classified as PNR (primary nonresponse) by the model, while those below the line are classified as PR (primary response) to infliximab therapy. The blue points represent the PR group confirmed by expert assessment; the red points then belong to the PNR group (VAT model, radiomics model based on features extracted from visceral adipose tissue; bowel model, radiomics model based on features extracted from the whole inflamed bowel; VAT-bowel model, a combination of the VAT model and bowel model)

**Fig. 5:**
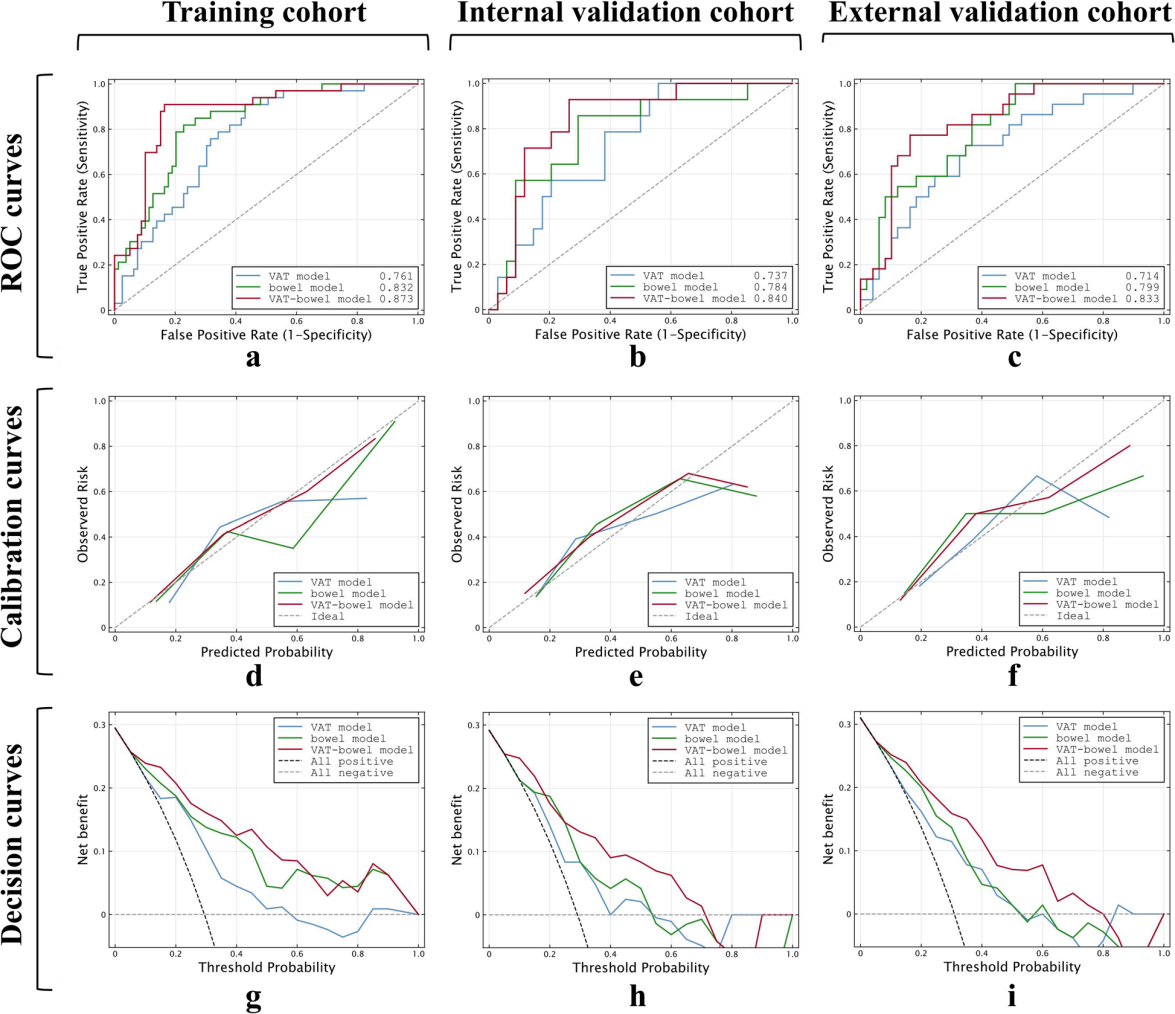
Predictive performance of VAT model, bowel model, and VAT-bowel model in the training cohort and internal and external validation cohorts. Plots in the first row are the ROC curves for the three models and show the performance to distinguish PNR from PR. The second row is the calibration curves of these three models in those three data cohorts, while plots in the third row are the results corresponding decision curve analysis (VAT model, radiomics model based on features extracted from visceral adipose tissue; bowel model, radiomics model based on features extracted from the whole inflamed bowel; VAT-bowel model, a combination of the VAT model and bowel model; ROC, receiver operating characteristic; AUC, area under the receiver operator characteristic curve; PNR, primary nonresponse; PR, primary response to infliximab therapy)

The VAT model alone could distinguish the PNR from PR group with a cut-off value of 0.280 (Fig. 4a and Table 3). The AUCs were 0.761 (95% CI, 0.672–0.839) in training cohort, 0.737 (95% CI, 0.590–0.854) in internal validation cohort, and 0.714 (95% CI, 0.595–0.815) in external validation cohort, respectively (all *p* < 0.005; Table 3; Fig. 5a–c). There were no significant differences in the AUCs among the three data cohorts according to DeLong’s test (all *p* > 0.500). With the Hosmer-Lemeshow test, the *χ*^2^ were 10.075 (*p* = 0.260), 13.76 (*p* = 0.088) and 2.056 (*p* = 0.979) in the training and two validation cohorts (Fig. 5d–f). VAT model possessed a relatively good net benefit in clinical utility over the three data cohorts, compared to the all positive and all negative curves (Fig. 5g–i).

**Table 3:** Predictive performance of radiomics models based on different features in differentiating PNR from PR in the training and validation cohorts

Variable	Accuracy	Sensitivity	Specificity	AUC (95% CI)	*p*
Training cohort (*PNR/PR* = 33/79)					
VAT radiomics model	0.705	0.727	0.696	0.761 (0.672–0.837)	< 0.001
Bowel radiomics model	0.732	0.848	0.684	0.832 (0.750–0.896)	< 0.001
VAT-bowel radiomics model	0.821	0.909	0.785	0.873 (0.797–0.928)	< 0.001
Internal validation cohort (*PNR/PR* = 14/34)					
VAT radiomics model	0.688	0.571	0.735	0.737 (0.590–0.854)	0.001
Bowel radiomics model	0.708	0.857	0.647	0.784 (0.641–0.889)	< 0.001
VAT-bowel radiomics model	0.813	0.714	0.853	0.840 (0.706–0.930)	< 0.001
External validation cohort (*PNR/PR* = 22/49)					
VAT radiomics model	0.662	0.727	0.633	0.714 (0.595–0.815)	0.001
Bowel radiomics model	0.690	0.590	0.735	0.799 (0.687–0.885)	< 0.001
VAT-bowel model	0.817	0.636	0.898	0.833 (0.726–0.911)	< 0.001

Accuracy, sensitivity, and specificity of the radiomic model in training and validation cohorts were calculated with the cut-off value of 0.280 (VAT radiomics model), 0.190 (bowel radiomics model), and 0.268 (VAT-bowel radiomics model), respectively, which maximizes the Youden index in the training cohort. *p* value is the significance level of comparison of AUC with that of random case (AUC = 0.5). *PNR*, primary nonresponse to infliximab therapy; *PR*, response to infliximab therapy. AUC, area under ROC curve; CI, confidence interval. VAT radiomics model, radiomics model based on the features extracted from visceral adipose tissue; bowel radiomics model, radiomics model based on the features extracted from the whole inflamed bowel; VAT-bowel radiomics model, a combination of the VAT and bowel radiomics models

### Development and validation of bowel and VAT-bowel radiomics models

Fourteen radiomics features were finally included in bowel model (Supplementary materials A7) with a cut-off vale of 0.190 (Fig. 4b). The bowel model reached a predictive performance to AUCs of 0.832 (95% CI, 0.750–0.896) in training cohort, 0.784 (95% CI, 0.641–0.889) in internal validation cohort, and 0.799 (95% CI, 0.687–0.885) in external validation cohort (all *p* < 0.001), respectively (Table 3; Fig. 5a–c).

**Fig. 6:**
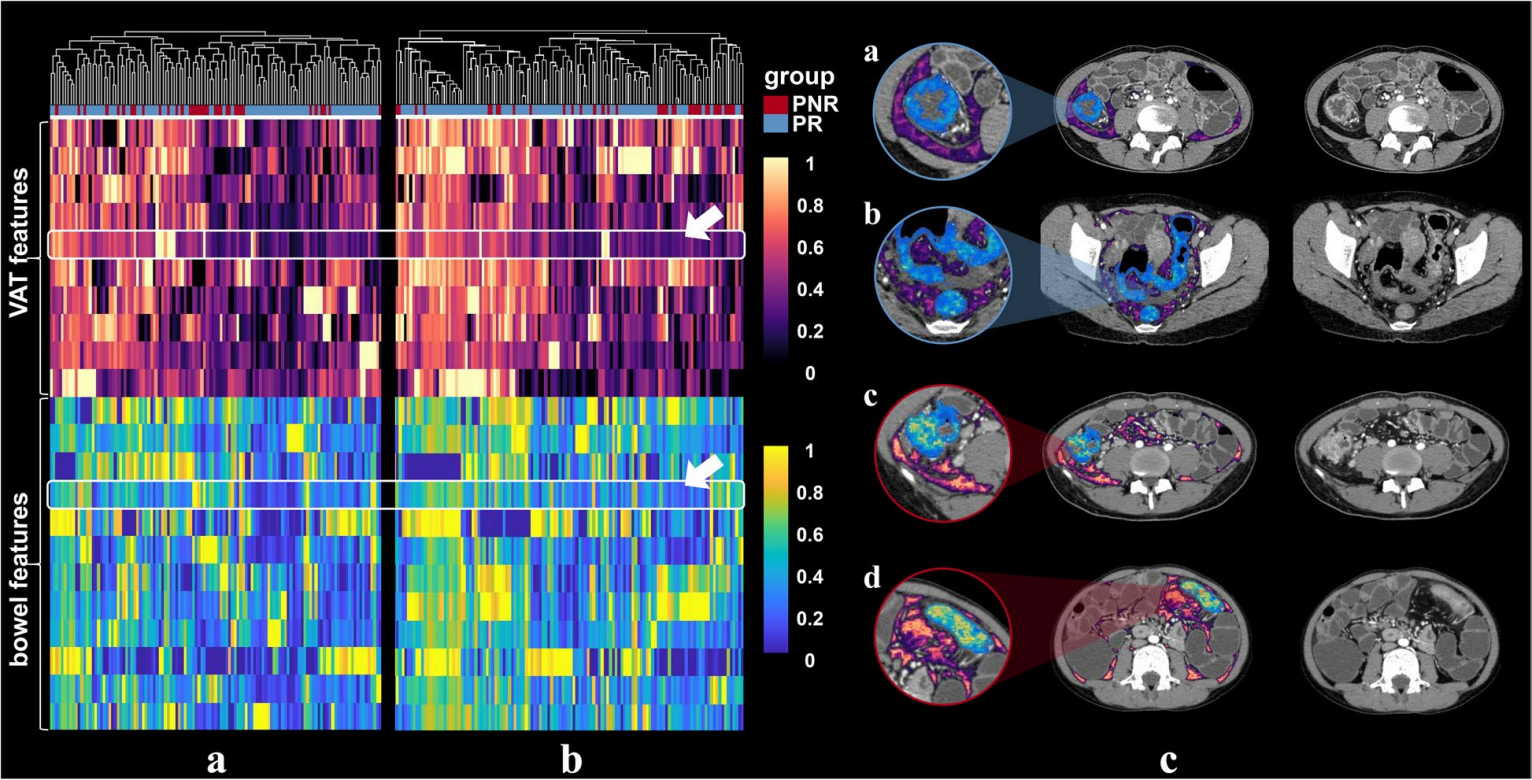
Heatmaps generated by unsupervised hierarchical clustering of the 22 selected features of VAT-bowel model in (**a**) training cohort and (**b**) the total validation cohort, and (**c**) examples of VAT and bowel feature maps overlaid on the CT images of four CD patients. The values of the heatmaps and feature maps are all standardized to the range of [0, 1], in order to achieve a clear view. In heatmaps (**a** and **b**), each row is one selected radiomics feature, and each column is a sample (PNR, red; PR, blue); the dendrogram at the top represents samples with similar information determined by clustering; the white arrows point to a VAT feature named “wavelet-LHH_glszm_LargeAreaLowGrayLevelEmphasis” or a bowel feature named “wavelet-LHL_glszm_LargeAreaEmphasis.” These two representative radiomics features are overlaid on CT images of four patients (cases a and b, with response to IFX therapy, predicted probabilities = 0.199 and 0.174; cases c and d, without response to IFX therapy, predicted probabilities = 0.622 and 0.523; VAT-bowel model’s cut-off value = 0.268) as shown in image (**c**). Both features demonstrate differences between patients in PR and PNR groups, with higher values from the PNR patients (cases c and d), suggesting more complex and coarse texture features of VAT and bowel. PNR, primary nonresponse; PR, primary response; IFX, infliximab; VAT, visceral adipose tissue; VAT-bowel model, radiomics model based on features extracted from VAT and the whole inflamed bowel

The finally selected 12 VAT radiomics features and 14 bowel radiomics features were combined for the development of VAT-bowel model, and 22 features with nonzero coefficients were subsequently retained from the total of 26 features according to LASSO with an optimal *λ* value of 0.006 (lnλ =  − 5.116; Supplementary Figure 2C; Supplementary Table 2 and Fig. 6a and b). The visualized radiomics feature maps (overlaid on CT images) of two important texture features extracted from VAT and bowel from two patients (1 PNR and 1 PR) were shown in Fig. 6c. The VAT-bowel model showed the best predicted power (Fig. 4c) with AUCs of 0.873 (95% CI, 0.797–0.928) in the training cohort, 0.840 (95% CI, 0.706–0.930) in the internal validation cohort, and 0.833 (95% CI, 0.726–0.911) in the external validation cohort (Table 3; Fig. 5a–c). However, no significant differences were found from the AUCs among the three data cohorts according to DeLong’s test (all *p* > 0.500).

### Predictive performance comparison between bowel and VAT-bowel radiomics models

The VAT-bowel model demonstrated superior performance over the bowel model for distinguishing PNR from PR, with higher AUC and accuracy in all data cohorts (Table 4 and Fig. 5). Although no significant differences were observed between the AUCs of the two models according to DeLong’s test (all *p* > 0.090), McNemar’s test revealed that the accuracy of the VAT-bowel model was significantly higher in training cohort (accuracy = 0.821 vs. 0.732, *p* = 0.076), internal validation cohort (accuracy = 0.813 vs. 0.708, *p* = 0.070), and external validation cohort (accuracy = 0.817 vs. 0.690, *p* = 0.035), which suggested good discrimination of VAT-bowel model.

**Table 4:** Predictive performance comparison of bowel and VAT-bowel radiomics models in differentiating PNR from PR in the training and validation cohorts

Variable	DeLong’s test		McNemar’s test		Hosmer-Lemeshow test		IDI	
	AUC (95% CI)	*P*^a^	Accuracy (95% CI)	*P*^b^	*χ*^2^	*P*^c^	Index (95% CI)	*P*^d^
Training cohort (*PNR/PR* = 33/79)								
Bowel radiomics model	0.832 (0.750–0.896)	0.165	0.732 (0.643–0.805)	0.076	11.056	0.199	0.031 (0.006–0.056)	0.016
VAT-bowel radiomics model	0.873 (0.797–0.928)		0.821 (0.740–0.881)		5.668	0.684		
Internal validation cohort (*PNR/PR* = 14/34)								
Bowel radiomics model	0.784 (0.641–0.889)	0.098	0.708 (0.568–0.818)	0.070	5.850	0.664	0.042 (0.005–0.078)	0.024
VAT-bowel radiomics model	0.840 (0.706–0.930)		0.813 (0.681–0.898)		10.08	0.259		
External validation cohort (*PNR/PR* = 22/49)								
Bowel radiomics model	0.799 (0.687–0.885)	0.376	0.690 (0.575–0.786)	0.035	9.139	0.331	0.037 (0.003–0.070)	0.032
VAT-bowel radiomics model	0.833 (0.726–0.911)		0.817 (0.711–0.890)		7.262	0.509		

*PNR* primary nonresponse to infliximab therapy, *PR* response to infliximab therapy. Bowel radiomics model, radiomics model based on the features extracted from the whole inflamed bowel; VAT-bowel radiomics model, radiomics model based on the features extracted from both visceral adipose tissue and the whole inflamed bowel. AUC, area under ROC curve; CI, confidence interval; IDI, integrated discriminant improvement. ^a^*p* value is the significance level of the comparison of AUC between the bowel and VAT-bowel radiomics models according to DeLong’s test. ^b^*p* value is the significance level of the comparison of accuracy between bowel and VAT-bowel radiomics models according to McNemar’s test. ^c^*p* value is the significance level of the difference between the prediction probability and the true value for each model according to Hosmer-Lemeshow test. ^d^*p* value is the significance level of the improvement (IDI > 0) in predictive performance of the new model (VAT-bowel model) over the old model (bowel model)

For calibration, both models exhibited good fit in all data cohorts with *p* > 0.100 by Hosmer-Lemeshow test (Table 4). The calibration curves of VAT-bowel model were closer to the ideal calibration curves than that of the bowel model as shown in Fig. 5d–f, indicating a relatively good calibration power of VAT-bowel model.

In addition, the IDI indices also indicated that the VAT-bowel model improved prediction efficacy compared to the bowel model in the training cohort (IDI = 0.031, *p* = 0.016), internal validation cohort (IDI = 0.042, *p* = 0.024), and external validation cohort (IDI = 0.037, *p* = 0.032).

With regard to the clinical utility, the decision curves (Fig. 5g–i) showed that the VAT-bowel model possessed a slightly better net benefit overall than the bowel model in predicting the outcome of IFX therapy.

## Discussion

In this study, we demonstrated that RM is able to capture the pathophysiological changes occurring in VAT. Based on features of RM, it is associated with the response to IFX and could potentially provide additional information in predicting therapeutic response. Furthermore, we developed a comprehensive radiomics model combining with VAT and bowel features. Compared to using bowel-RM alone, this integrated model yielded significant improvement in predictive ability with the IDI of 0.031 in training cohort and 0.042 and 0.037 in two independent validation cohorts, respectively (all *p* < 0.05).

Current knowledge on the use of VAT in CD remains limited. Numerous studies have already indicated a positive correlation between VAT and CD activity, as well as its ability to predict complications, recurrence, and suboptimal response to biologic therapies, despite the mechanisms remaining elusive [12]. The accumulating evidence suggests that the metabolically active visceral adipose compartment may serve as a possible source of proinflammatory substances [12, 21]. BMI was used to assess VAT. However, it was not suitable for CD patients because of the malnutrition-induced weight lost [22, 23]. The quantitative VAT analysis with manually outlined contours from several vertebral levels can fail to capture microstructure with important messages in disease progress, although it has been used as indicators in predicting treatment efficacy [24, 25]. Moreover, time-consuming deficiencies in computing the area hinder its clinical applicability.

The mechanism underlying the correlation between adipose and inflammation of CD remains elusive. Studies have shown that adipose tissue plays a crucial role in the production of proinflammatory cytokines such as tumor necrosis factor-alpha (TNFα), interleukin-(IL)-6 (IL-6), and IL-8 (CXCL8) [26]. The presence of VAT is believed to establish a responsive immunological region surrounding the irritated intestine. The maintenance of balance between the host’s immune system and commensal microbiota heavily relies on the integrity of the gastrointestinal epithelial barrier. The translocation of bacteria into the mesenteric tissue leads to the development of mesenteric adipose and chronic inflammation, resulting in subsequent mucosal damage and inflammation [26]. High visceral adipose predicts complicated CD and disease exacerbation [24]. Our previous study also substantiates the utility of VAT as an indicator for assessing disease severity [9]. Therefore, we think that the VAT of CD expressing higher amounts of TNF-α can also affect the response to infliximab in patients with CD and may be acted as image features.

With the advent of medical artificial intelligence, it enables the comprehensive extraction of multidimensional information from lesions. Radiomics quantifies image features using voxel values and their interrelationships. In our study, morphological features such as flatness and sphericity provide a quantitative description of the physical appearance of the lesion. It is worth to note that 88.9% (10/12) of the VAT features were wavelet. The wavelet transform enables the decomposition of noise and useful signal into different scales, allowing for the conversion of wavelet coefficients and thereby facilitating the distinction between useful signal and noise [27]. CD patients showed different VAT texture characteristics from healthy people’s CT imaging. In medical images, the quantitative or qualitative changes of texture features often reflect the pathological changes of the body. Besides, it was reported that wavelet features were strongly associated with survival in patients with hepatocellular carcinoma and biological characteristics of ICC, which can also quantify intratumoral heterogeneity [28, 29]. The wavelet transformation offers possibility to decompose special patterns, not visible to the naked eye, and enables the quantification of VAT heterogeneity, was caused from pathological variation or inflammatory cytokine infiltrations [30, 31]. Our study substantiated that VAT-RM can effectively capture these pathological changes in VAT in CD noninvasively and convert them into radiomic features, thereby reinforcing the evidence of mechanisms to impact VAT on revealing therapeutic efficacy and influencing strategy selection. However, it is also challenging to infer the connection between these characteristics and biological differences solely from the data presented in this study, thus necessitating additional research on the underlying factors contributing to variations in radiomics features within adipose tissue.

Consistent with previous study [17], bowel RM served as a promising technique in tailoring treatment strategy in CD patients, exhibiting satisfactory performance in predicting effectiveness and robustness, while peri-lesion microenvironment such as VAT was ignored [17]. In our study, the combined model consisting of VAT and bowel radiomics features outperformed bowel RM alone for identifying CD patients at high risk of PNR for IFX both in training and testing cohort. From a statistical point of view, although the lack of a significant improvement in AUCs suggests that the overall performances of the two models are roughly equivalent, it is worth noting that the accuracies of the VAT-bowel RM tend to be higher at the chosen threshold, and the variations in the IDIs for the integrated model are meaningful. Moreover, VAT-bowel RM exhibits better goodness of fit and overall has a slightly better net benefit than bowel RM alone. Our study showed that the comprehensive model was superior to the bowel RM alone, which could provide more information to judge the probability of achieving PNR before treatment in patient who intends to receive IFX treatment.

This study had several limitations. Although, MRE is a preferred examination for CD patients as it is radiative-free and can provide more biological information [32], we used CTE rather than magnetic resonance enterography (MRE) to develop the radiomic signature. In future prospective studies, however, a CT-based radiomics framework may facilitate artificial development in the field of MR through transfer learning. Secondly, the radiomics signatures were extracted from single-phase CT images, underutilizing the information in the CT images. We will integrate the radiomic information from plain, arterial, and venous CT images in further research. Lastly, the sample size in this study is still limited. Multicenter validation with a larger sample size of patients is essential to obtain higher-level evidence for future clinical applications.

In conclusion, VAT has effect on detection of IFX treatment response and improves the performance for identification of CD patients at high risk of primary nonresponse to IFX therapy. We have conducted a CT-based radiomics model (RM), composed from influencing factors for VAT-bowel analysis in differentiation nonresponse from response patients under IFX treatment. Our results suggested that comprehensive RM captured the pathological changes occurring in VAT and bowel lesions, which could help to identify CD patients who will be resistant to IFX at the beginning of therapy.

## Supplementary Information

**Additional file 1: A1. **Volume of interest segmentation.** A2. **Feature extraction. **A3.** Radiomics features extraction and selection. **A4.** Binary classification model building for distinguishing between PR and PNR by a support vector machine classifier. **A5.** Evaluation of sample size. A6. Radiomics features extraction and selection. **A7.** Feature extraction and selection result of the bowel radiomics model. **Supplementary Table 1.** The selected radiomics features of the bowel radiomics model and the corresponding coefficients. **Supplementary Table 2.** The selected radiomics features of the VAT-bowel radiomics model for identifying PNR from PR to infliximab therapy. **Supplement Figure 1.** Inclusion and exclusion criteria and recruitment pathways for patients in this study. (CD, Crohn's disease; IFX, infliximab; PR, primary response; PNR, primary nonresponse; Centre 1, The First Affiliated Hospital of Sun Y at-Sen University; Centre 2, The Sixth Affiliated Hospital of Sun Yat-Sen University). **Supplementary Figure 2.** LASSO coefficient profile plots of the selected radiomics features in (A) VAT model, (B) bowel model and (C) VAT-bowel model. In each plot, the x-axis at the bottom represents , while the x-axis at the top is the number of the rest radiomics features that vary with lambda. The vertical dashed line indicates the optimal lambda value [= -3.381, -3.194 and-5.116, respectively], resulting in 12, 14 and 22 radiomics features with non-zero coefficients for each model finally. (LASSO, least absolute shrinkage and selection operator; VAT model, radiomics model based on features extracted from visceral adipose tissue; bowel model, radiomics model based on features extracted from the whole inflamed bowel; VAT-bowel model, a combination of the VAT model and bowel model).

## Data Availability

The data that support the findings of this study are available from the corresponding authors with a signed data access agreement. The raw image data are not publicly available because they contain sensitive information that could compromise patient privacy. We are pleased to share all the codes for feature extraction and model construction upon request for research purpose.

## Abbreviations

AUC: Area under the curve
CD: Crohn’s disease
CI: Confidence interval
CTE: Computed tomography enterography
IBD: Inflammatory bowel disease
IDI: Integrated differentiation improvement
IFX: Infliximab
LASSO: Least absolute shrinkage and selection operator
LOOCV: Leave-one-out cross validation
MRE: Magnetic resonance enterography
PNR: Primary nonresponse
PR: Primary response
RM: Radiomics model
ROC: Receiver operating characteristic
SES-CD: Simple endoscopic score for Crohn’s disease
SVM: Support vector machine
TNF-α: Targeting tumor necrosis factor-α
VAT: Visceral adipose tissue

## References

[1] Feld L, Glick LR, Cifu AS (2019) Diagnosis and management of Crohn disease. JAMA 321(18):1822–1823 30969326 10.1001/jama.2019.3684

[2] Ding NS, Hart A, De Cruz P (2016) Systematic review: predicting and optimising response to anti-TNF therapy in Crohn’s disease - algorithm for practical management. Aliment Pharmacol Ther 43(1):30–51 26515897 10.1111/apt.13445

[3] Dotan I, Ron Y, Yanai H et al (2014) Patient factors that increase infliximab clearance and shorten half-life in inflammatory bowel disease: a population pharmacokinetic study. Inflamm Bowel Dis 20(12):2247–2259 25358062 10.1097/MIB.0000000000000212

[4] Fischer S, Neurath MF (2017) Precision medicine in inflammatory bowel diseases. Clin Pharmacol Ther 102(4):623–632 28699158 10.1002/cpt.793

[5] Sprakes MB, Ford AC, Warren L, Greer D, Hamlin J (2012) Efficacy, tolerability, and predictors of response to infliximab therapy for Crohn’s disease: a large single centre experience. J Crohns Colitis 6(2):143–153 22325168 10.1016/j.crohns.2011.07.011

[6] Wong U, Cross RK (2017) Primary and secondary nonresponse to infliximab: mechanisms and countermeasures. Expert Opin Drug Metab Toxicol 13(10):1039–1046 28876147 10.1080/17425255.2017.1377180

[7] Papamichael K, Gils A, Rutgeerts P et al (2015) Role for therapeutic drug monitoring during induction therapy with TNF antagonists in IBD: evolution in the definition and management of primary nonresponse. Inflamm Bowel Dis 21(1):182–197 25222660 10.1097/MIB.0000000000000202

[8] Gillies RJ, Kinahan PE, Hricak H (2016) Radiomics: images are more than pictures, they are data. Radiology 278(2):563–577 26579733 10.1148/radiol.2015151169 PMC4734157

[9] Li XH, Feng ST, Cao QH et al (2021) Degree of creeping fat assessed by computed tomography enterography is associated with intestinal fibrotic stricture in patients with Crohn’s disease: a potentially novel mesenteric creeping fat index. J Crohns Colitis 15(7):1161–1173 33411893 10.1093/ecco-jcc/jjab005 PMC8427713

[10] Bryant RV, Schultz CG, Ooi S et al (2019) Visceral adipose tissue is associated with stricturing Crohn’s disease behavior, fecal calprotectin, and quality of life. Inflamm Bowel Dis 25(3):592–600 30215805 10.1093/ibd/izy278

[11] Holt DQ, Moore GT, Strauss BJ et al (2017) Visceral adiposity predicts post-operative Crohn’s disease recurrence. Aliment Pharmacol Ther 45(9):1255–1264 28244124 10.1111/apt.14018

[12] Eder P, Adler M, Dobrowolska A, Kamhieh-Milz J, Witowski J (2019) The role of adipose tissue in the pathogenesis and therapeutic outcomes of inflammatory bowel disease. Cells 8(6):628 10.3390/cells8060628 PMC6627060 31234447

[13] Bilski J, Mazur-Bialy A, Wojcik D et al (2019) Role of obesity, mesenteric adipose tissue, and adipokines in inflammatory bowel diseases. Biomolecules 9(12) 10.3390/biom9120780 PMC6995528 31779136

[14] Wang Z, Meng Y, Weng F et al (2020) An effective CNN method for fully automated segmenting subcutaneous and visceral adipose tissue on CT scans. Ann Biomed Eng 48(1):312–328 31451989 10.1007/s10439-019-02349-3

[15] Gu P, Chhabra A, Chittajallu P et al (2022) Visceral adipose tissue volumetrics inform odds of treatment response and risk of subsequent surgery in IBD patients starting antitumor necrosis factor therapy. Inflamm Bowel Dis 28(5):657–666 34291800 10.1093/ibd/izab167

[16] Tabari A, Kilcoyne A, Jeck WR, Mino-Kenudson M, Gee MS (2019) Texture analysis of magnetic resonance enterography contrast enhancement can detect fibrosis in Crohn disease strictures. J Pediatr Gastroenterol Nutr 69(5):533–538 31365485 10.1097/MPG.0000000000002454

[17] Li X, Zhong Y, Yuan C et al (2022) Identifying patients with Crohn’s disease at high risk of primary nonresponse to infliximab using a radiomic-clinical model. Int J Intelligent Syst 37(12):11853–11870

[18] Khanna R, Ma C, Jairath V et al (2022) Endoscopic assessment of inflammatory bowel disease activity in clinical trials. Clin Gastroenterol Hepatol 20(4):727–736.e2 33338657 10.1016/j.cgh.2020.12.017

[19] Danese S, Sandborn WJ, Colombel JF et al (2019) Endoscopic, radiologic, and histologic healing with vedolizumab in patients with active Crohn’s disease. Gastroenterology 157(4):1007–1018.e7 31279871 10.1053/j.gastro.2019.06.038

[20] Isensee F, Jaeger PF, Kohl SAA, Petersen J, Maier-Hein KH (2021) nnU-Net: a self-configuring method for deep learning-based biomedical image segmentation. Nat Methods 18(2):203–211 33288961 10.1038/s41592-020-01008-z

[21] Kredel L, Batra A, Siegmund B (2014) Role of fat and adipokines in intestinal inflammation. Curr Opin Gastroenterol 30(6):559–565 25188546 10.1097/MOG.0000000000000116

[22] Kurnool S, Nguyen NH, Proudfoot J et al (2018) High body mass index is associated with increased risk of treatment failure and surgery in biologic-treated patients with ulcerative colitis. Aliment Pharmacol Ther 47(11):1472–1479 29665045 10.1111/apt.14665 PMC5992082

[23] Hu Q, Ren J, Li G, Wu X, Li J (2017) The impact of obesity on the clinical course of inflammatory bowel disease: a meta-analysis. Med Sci Monit 23:2599–2606 28552901 10.12659/MSM.901969 PMC5461885

[24] Shen W, Cao L, Li Y et al (2018) Visceral fat is associated with mucosal healing of infliximab treatment in Crohn’s disease. Dis Colon Rectum 61(6):706–712 29722729 10.1097/DCR.0000000000001074

[25] Connelly TM, Juza RM, Sangster W et al (2014) Volumetric fat ratio and not body mass index is predictive of ileocolectomy outcomes in Crohn’s disease patients. Dig Surg 31(3):219–224 25277149 10.1159/000365359

[26] Fink C, Karagiannides I, Bakirtzi K, Pothoulakis C (2012) Adipose tissue and inflammatory bowel disease pathogenesis. Inflamm Bowel Dis 18(8):1550–1557 22407798 10.1002/ibd.22893 PMC3374883

[27] Kyriacos U, Burger D, Jordan S (2019) Testing effectiveness of the revised Cape Town modified early warning and SBAR systems: a pilot pragmatic parallel group randomised controlled trial. Trials 20(1):809 31888745 10.1186/s13063-019-3916-0 PMC6937946

[28] Chen S, Zhu Y, Liu Z, Liang C (2017) Texture analysis of baseline multiphasic hepatic computed tomography images for the prognosis of single hepatocellular carcinoma after hepatectomy: a retrospective pilot study. Eur J Radiol 90:198–204 28583634 10.1016/j.ejrad.2017.02.035

[29] Ma X, Qian X, Wang Q et al (2023) Radiomics nomogram based on optimal VOI of multi-sequence MRI for predicting microvascular invasion in intrahepatic cholangiocarcinoma. Radiol Med 128(11):1296–1309 10.1007/s11547-023-01704-8 PMC10620280 37679641

[30] Sarioglu O, Sarioglu FC, Capar AE et al (2022) Clot-based radiomics features predict first pass effect in acute ischemic stroke. Interv Neuroradiol 28(2):160–168 34000866 10.1177/15910199211019176 PMC9131494

[31] Zwanenburg A, Vallières M, Abdalah MA et al (2020) The image biomarker standardization initiative: standardized quantitative radiomics for high-throughput image-based phenotyping. Radiology 295(2):328–338 32154773 10.1148/radiol.2020191145 PMC7193906

[32] Cushing K, Higgins PDR (2021) Management of Crohn disease: a review. JAMA 325(1):69–80 33399844 10.1001/jama.2020.18936 PMC9183209

