# Supplementary material for: CT-based radiomics signature of visceral adipose tissue and bowel lesions for identifying patients with Crohn’s disease resistant to infliximab
Source: Insights Imaging. 2024 Jan 30;15:28. doi: 10.1186/s13244-023-01581-9 (PMC10828370; doi:10.1186/s13244-023-01581-9)
Supplement: Supplementary file 1 — Additional file 1: A1. Volume of interest segmentation. A2. Feature extraction. A3. Radiomics features extraction and selection. A4. Binary classification model building for distinguishing between PR and PNR by a support vector machine classifier. A5. Evaluation of sample size. A6. Radiomics features extraction and selection. A7. Feature extraction and selection result of the bowel radiomics model. Supplementary Table 1. The selected radiomics features of the bowel radiomics model and the corresponding coefficients. Supplementary Table 2. The selected radiomics features of the VAT-bowel radiomics model for identifying PNR from PR to infliximab therapy. Supplement Figure 1. Inclusion and exclusion criteria and recruitment pathways for patients in this study. (CD, Crohn's disease; IFX, infliximab; PR, primary response; PNR, primary nonresponse; Centre 1, The First Affiliated Hospital of Sun Y at-Sen University; Centre 2, The Sixth Affiliated Hospital of Sun Yat-Sen University). Supplementary Figure 2. LASSO coefficient profile plots of the selected radiomics features in (A) VAT model, (B) bowel model and (C) VAT-bowel model. In each plot, the x-axis at the bottom represents , while the x-axis at the top is the number of the rest radiomics features that vary with lambda. The vertical dashed line indicates the optimal lambda value [= -3.381, -3.194 and-5.116, respectively], resulting in 12, 14 and 22 radiomics features with non-zero coefficients for each model finally. (LASSO, least absolute shrinkage and selection operator; VAT model, radiomics model based on features extracted from visceral adipose tissue; bowel model, radiomics model based on features extracted from the whole inflamed bowel; VAT-bowel model, a combination of the VAT model and bowel model). [file 13244_2023_1581_MOESM1_ESM.docx]

**CT-based radiomics signature of visceral adipose tissue and bowel lesions for identifying patients with Crohn’s disease resistant to infliximab**

**ELECTRONIC SUPPLEMENTARY MATERIAL**

**A1. Volume of interest segmentation**

The training set of the model included 10 samples randomly selected from the training cohort, each of which was meticulously outlined the contours of all visceral fat tissue by a radiologist (Z.R. with 5 years of experience in abdominal CT, and Y.W.) as the reference standard of the VAT segmentation task. The segmentation model was trained in the Python environment (version 3.6; https://www.python.org/) using PyTorch (version 1.9.0; https://pytorch.org/).

The mean dice similarity coefficient for VAT reached 0.960 (calculated by the reference standard and automatic segmentation results of VAT), demonstrated an excellent performance of the automatic segmentation. As a result, the VAT of the remaining samples in the training and validation cohorts were automatically segmented by the trained model. Afterwards, a radiologist (Y.W.) scrutinized and modified the segmentation results of VAT on CT images with the open-source software ITK-SNAP (version 3.4.0; www.itksnap.org) to determine the final volume of interest (VOI).

**A2. Feature extraction**

For each volume of interest (VOI), filter “Laplacian of Gaussian (LoG)” with three different sigma values (1.0, 3.0 and 5.0) and filter “Wavelet” with eight different filtering patterns (high-pass[H] and low-pass[L] filters combined in x, y and z axes, resulting in HHH, HHL, HLH, HLL, LHH, LHL, LLH and LLL filters) were performed on the original image (12 images in total). For each image, 18 first-order features and 75 texture features were calculated, resulting in (18+75)×12=1116 features. Additionally, 14 shape features that assessed the spatial properties of three-dimensional VOI were extracted from original images. Therefore, 1116+14=1130 radiomics features were available for each VOI in total.

Additionally, the extracted texture features included 24 Gray Level Co-occurrence Matrix (GLCM) features (e.g., Correlation or Contrast), 16 Gray Level Size Zone Matrix (GLSZM) features (e.g., Small Area Emphasis or Zone Variance), 16 Gray Level Run Length Matrix (GLRLM) features (e.g., Gray Level Variance or Gray Level Non-Uniformity), 14 Gray Level Dependence Matrix (GLDM) features (e.g., Dependence Non-Uniformity or High Gray Level Emphasis), and 5 Neighbourhood Gray Tone Difference Matrix (NGTDM) features (e.g., Complexity or Strength).

**A3. Radiomics features extraction and selection**

To investigate the reproducibility of the extracted features, inter- and intra-observer analysis was performed and intraclass correlation coefficients (ICCs) were calculated on 30 samples randomly selected from the training cohort. Two radiologists performed additional VOI modifications using the same tool and environment settings. The time interval between two readings by the radiologist was over 3 months for intra-observer analysis. Only radiomics features with excellent robustness (ICC ≥ 0.9) were included in the subsequent analysis.

To further purify features, we conducted Z-score normalization on the features to minimize the impact of the dimensional differences in the data, and then applied the least absolute shrinkage and selection operator (LASSO) algorithm to select features with non-zero coefficients. This procedure was executed on the training cohort and used for the validation cohorts, implementing in R (version 4.0.4; *http://www.r-project.org/*).**A4. Binary classification model building for distinguishing between PR and PNR by a support vector machine classifier**

In order to determine the optimal parameters for the Support Vector Machine (SVM) classifier, the leave-one-out cross validation (LOOCV) strategy was adopted in the training cohort, and a grid search method was utilized to identify the following hyper-parameters: *kernel function* (linear/polynomial/radial basis function/sigmoid), *C* (regularization parameter), *max iter* (hard limit on iterations within solver), and *gamma* (kernel coefficient).

**A5. Evaluation of sample size**

A sample size of 48 patients (34 PR and 14 PNR) is required for the LOOCV modeling of IFX therapy prediction based on the following conditions by using MedCalc Statistical Software (version 15.8; [*http://www.medcalc.org/*):](http://www.medcalc.org/):) power, 80%; a two-tailed significance level, 0.05; alternative hypothesis of the area under the receiver operating characteristic (ROC) curve (AUC), 0.75; the null hypothesis of AUC=0.5; ratio of classes, the real ratio of sample sizes in the negative and positive groups (training cohort = 79 PR / 33 PNR). Therefore, the sample size for modeling in this study was considered able to detect an AUC over 0.75 different from 0.5 with 80% power.

**A6. Radiomics features extraction and selection**

According to the inter- and intra-observer analysis, 1119 features with ICCs ≥ 0.9 were retained. Subsequently, 12 features with non-zero coefficients were finally selected according to LASSO. The correlation coefficients of the features selected by LASSO algorithm are shown in Table 2, and the heatmap of selected features in training and validation cohorts are presented in Figure 3A and B, respectively.

**A7. Feature extraction and selection result of the bowel radiomics model**

The VOI segmentation, feature extraction and selection procedure were completely consistent with our previous study[1]. According to inter- and intra-observer analysis, 889 radiomics features with intraclass correlation coefficients (ICCs) ≥ 0.9 were retained among the 1130 radiomics features extracted from each volume of interest (VOI) of the inflamed bowel. Subsequently, 14 features with non-zero coefficients were finally selected according to the least absolute shrinkage and selection operator (LASSO) algorithm with an optimal lambda ($\lambda$) value of 0.041 ($\ln\lambda=-3.194$; Supplementary figure 2B). The correlation coefficients of those 14 selected features are shown in Supplementary table 1.

**Supplementary table**

**Supplementary table 1. The selected radiomics features of the bowel radiomics model and the corresponding coefficients**

| Feature’s name | Coefficient  (absolute value) |
| --- | --- |
| wavelet-HLH_firstorder_Energy | 0.0924 |
| wavelet-LHL_glszm_LargeAreaEmphasis | 0.0763 |
| wavelet-HHL_glszm_SizeZoneNonUniformity | 0.0275 |
| original_gldm_SmallDependenceHighGrayLevelEmphasis | 0.0173 |
| wavelet-HHH_glszm_LowGrayLevelZoneEmphasis | 0.0158 |
| wavelet-LLL_gldm_DependenceVariance | 0.0146 |
| wavelet-HHH_glszm_ZoneVariance | 0.0121 |
| wavelet-LHL_glrlm_LongRunEmphasis | 0.0084 |
| wavelet-LHH_glrlm_ShortRunLowGrayLevelEmphasis | 0.0068 |
| log-sigma-3-0-mm-3D_ngtdm_Contrast | 0.0040 |
| log-sigma-1-0-mm-3D_ngtdm_Contrast | 0.0032 |
| wavelet-LLL_gldm_SmallDependenceHighGrayLevelEmphasis | 0.0024 |
| wavelet-LHL_glszm_ZoneVariance | 0.0018 |
| wavelet-LHH_glszm_LargeAreaHighGrayLevelEmphasis | 0.0002 |

Note. The bowel radiomics model was developed based on the features extracted from the inflamed bowel.

The Coefficient of each radiomics feature was generated by the least absolute shrinkage and selection operator algorithm, and presented as absolute value.

Each feature was named by concatenating the image type from which the feature was extracted, feature group and feature name by underline. For example, wavelet-LHL_glszm_LargeAreaEmphasis was a feature extracted from the image transformed by wavelet filter with HHH filtering pattern, gray level size zone matrix (glszm) in the texture group and the feature name was LargeAreaEmphasis.

Glszm, gray level size zone matrix; gldm, gray level dependence matrix; ngtdm, neighbourhood gray tone difference matrix; glcm, gray level co-occurrence matrix; all features above belong to texture features.

**Supplementary table 2. The selected radiomics features of the VAT-bowel radiomics model for identifying PNR from PR to infliximab therapy**

| VOI | Radiomics feature | Coefficient | Training Cohort | | | Internal Validation Cohort | | | External Validation Cohort | | |
| --- | --- | --- | --- | --- | --- | --- | --- | --- | --- | --- | --- |
|  |  |  | PR | PNR | *P** | PR | PNR | *P** | PR | PNR | *P** |
| Visceral Adipose Tissue | wavelet-HHH_glrlm_ShortRunLowGrayLevelEmphasis | 0.092 | -0.172  (-0.256, -0.101) | -0.172  (-0.212, -0.132) | 0.970 | -0.188  (-0.743, -0.122) | -0.201  (-0.228, -0.172) | 0.400 | -0.512  (-0.644, 0.524) | 0.448  (-0.582, 0.489) | 0.750 |
|  | original_shape_Flatness | -0.091 | -0.119  (-0.692, 0.498) | 0.231  (-0.117, 1.192) | 0.003 | 0.022  (-0.675, 0.455) | 0.272  (-0.145, 0.816) | 0.121 | -0.228  (-0.702, 0.775) | 0.015  (-0.264, 0.525) | 0.218 |
|  | wavelet-HHL_glcm_MCC | 0.076 | -0.166  (-0.623, 0.458) | -0.129  (-0.752, 0.820) | 0.804 | 0.044  (-0.384, 0.608) | -0.447  (-1.029, 0.030) | 0.021 | -0.068  (-0.467, 0.593) | -0.302  (-1.078, 0.164) | 0.132 |
|  | original_shape_Sphericity | 0.054 | 0.133  (-0.504, 0.723) | -0.469  (-0.914, 0.603) | 0.078 | -0.074  (-0.813, 0.624) | -0.235  (-0.415, 0.538) | 0.652 | -0.251  (-0.646, 0.507) | -0.349  (-0.751, -0.012) | 0.354 |
|  | wavelet-LHH_glszm_LargeAreaLowGrayLevelEmphasis | 0.041 | -0.399  (-0.449, -0.189) | -0.267  (-0.440, 0.367) | 0.069 | -0.392  (-0.455, -0.023) | -0.153  (-0.440, 0.560) | 0.090 | -0.473  (-0.750, 0.177) | -0.163  (-0.620, 0.577) | 0.064 |
|  | wavelet-LLH_firstorder_Minimum | 0.025 | 0.218  (-0.473, 0.719) | -0.065  (-0.592, 0.733) | 0.421 | 0.220  (-1.149, 0.683) | 0.350  (-0.752, 0.959) | 0.518 | 0.316  (-0.006, 0.618) | 0.062  (-0.545, 0.300) | 0.039 |
|  | wavelet-HHH_firstorder_Skewness | -0.016 | -0.128  (-0.501, 0.448) | 0.250  (-0.229, 0.608) | 0.078 | 0.118  (-0.068, 0.264) | 0.041  (-0.148, 0.422) | 0.669 | -0.119  (-0.473, 0.330) | -0.135  (-0.631, 0.278) | 0.873 |
|  | wavelet-LHL_glrlm_LongRunLowGrayLevelEmphasis | -0.015 | -0.220  (-0.732, 0.232) | 0.037  (-0.482, 0.502) | 0.085 | -0.431  (-0.777, 0.234) | 0.465  (-0.202, 1.105) | 0.004 | -0.323  (-0.751, 0.357) | 0.197  (-0.244, 1.083) | 0.052 |
|  | wavelet-LHL_glcm_JointEnergy | 0.007 | 0.141  (-0.407, 0.685) | -0.111  (-0.931, 0.480) | 0.134 | 0.304  (-0.416, 1.079) | 0.014  (-0.515, 0.379) | 0.086 | 0.312  (-1.600, 0.942) | -0.675  (-1.774, -0.125) | 0.015 |
|  | wavelet-HLL_glcm_InverseVariance | -0.003 | 0.312  (-0.518, 0.603) | 0.192  (-0.992, 0.674) | 0.495 | 0.055  (-0.241, 0.578) | -0.262  (-1.595, 0.609) | 0.100 | 0.190  (-0.418, 0.736) | -0.232  (-0.592, 0.133) | 0.102 |
| Bowel | wavelet-HLH_firstorder_Energy | -0.111 | -0.428  (-0.877, 0.201) | 0.140  (-0.207, 1.097) | 0.002 | -0.308  (-0.864, 0.347) | 0.247  (-0.299, 0.708) | 0.055 | -0.499  (-1.055, -0.017) | 0.855  (-0.123, 1.718) | <0.001 |
|  | wavelet-HHL_glszm_SizeZoneNonUniformity | 0.087 | -0.334  (-0.436, -0.067) | -0.293  (-0.356, -0.059) | 0.360 | -0.333  (-0.441, -0.073) | -0.305  (-0.375, -0.138) | 0.397 | -0.480  (-0.513, -0.413) | -0.388  (-0.462, -0.302) | <0.001 |
|  | original_gldm_SmallDependenceHighGrayLevelEmphasis | 0.065 | -0.316  (-0.361, -0.111) | -0.294  (-0.355, -0.126) | 0.709 | -0.297  (-0.363, 0.201) | -0.270  (-0.334, -0.215) | 0.469 | -0.286  (-0.330, -0.121) | -0.173  (-0.269, 0.063) | 0.021 |
|  | wavelet-LHL_glszm_LargeAreaEmphasis | -0.048 | -0.427  (-0.697, -0.091) | 0.034  (-0.241, 0.725) | <0.001 | -0.496  (-0.623, -0.075) | 0.366  (-0.308, 0.729) | 0.012 | -0.657  (-0.726, -0.490) | -0.411  (-0.619, -0.104) | <0.001 |
|  | wavelet-HHH_glszm_LowGrayLevelZoneEmphasis | 0.043 | -0.134  (-0.963, 1.030) | -0.910  (-1.088, 0.966) | 0.051 | 0.437  (-0.995, 1.051) | -0.713  (-1.012, 1.004) | 0.549 | 0.141  (-0.793, 1.054) | -0.909  (-1.033, 0.006) | 0.006 |
|  | log-sigma-3-0-mm-3D_ngtdm_Contrast | 0.027 | -0.462  (-0.901, 0.189) | 0.225  (-0.193, 0.874) | <0.001 | -0.395  (-0.858, 0.375) | 0.427  (-0.334, 0.702) | 0.060 | -0.925  (-1.174, -0.691) | -0.364  (-0.767, 0.200) | <0.001 |
|  | wavelet-LHH_glszm_LargeAreaHighGrayLevelEmphasis | -0.027 | -0.547  (-0.802, -0.147) | 0.022  (-0.300, 1.778) | <0.001 | -0.629  (-0.774, -0.119) | 0.109  (-0.466, 1.409) | 0.004 | -0.818  (-0.862, -0.721) | -0.660  (-0.791, -0.331) | <0.001 |
|  | wavelet-HHH_glszm_ZoneVariance | -0.022 | -0.482  (-0.669, -0.140) | -0.134  (-0.418, 0.745) | <0.001 | -0.452  (-0.664, -0.121) | 0.215  (-0.164, 1.125) | 0.004 | -0.622  (-0.735, -0.395) | -0.257  (-0.451, 0.068) | <0.001 |
|  | wavelet-LHH_glrlm_ShortRunLowGrayLevelEmphasis | 0.012 | -0.390  (-0.654, -0.030) | -0.116  (-0.523, 0.411) | 0.068 | -0.403  (-0.635, 0.403) | -0.158  (-0.444, 0.227) | 0.207 | -0.293  (-0.663, 0.610) | 1.085  (0.276, 2.416) | <0.001 |
|  | wavelet-LLL_gldm_SmallDependenceHighGrayLevelEmphasis | -0.012 | 0.155  (-0.611, 0.569) | 0.417  (0.091, 0.904) | 0.009 | -0.142  (-0.916, 0.297) | 0.243  (-0.003, 0.950) | 0.011 | -0.534  (-2.570, 0.171) | -0.055  (-0.571, 0.318) | 0.045 |
|  | log-sigma-1-0-mm-3D_ngtdm_Contrast | 0.011 | 0.105  (-0.895, 0.925) | 0.401  (-0.010, 0.994) | 0.064 | -0.073  (-0.888, 0.697) | 0.265  (-0.031, 1.191) | 0.273 | 0.230  (-0.435, 0.593) | 0.612  (0.373, 0.808) | 0.007 |
|  | wavelet-LHL_glszm_ZoneVariance | -0.001 | -0.207  (-0.515, 0.064) | -0.217  (-0.502, 0.210) | 0.898 | -0.377  (-0.611, 0.122) | -0.094  (-0.374, 0.315) | 0.131 | -0.344  (-0.529, -0.092) | -0.201  (-0.479, -0.138) | 0.291 |

Note. The VAT-bowel radiomics model was developed based on the features extracted from visceral adipose tissue and the inflamed bowel.

PNR, primary nonresponse (to infliximab therapy); PR, primary response (to infliximab therapy); VOI, volume of interest.

Glrlm, gray level run length matrix; glcm, gray level co-occurrence matrix; glszm, gray level size zone matrix; gldm, gray level dependence matrix; all features above belong to texture features.

Each feature was named by concatenating the image type from which the feature was extracted, feature group and feature name by underline. For example, original_shape_Flatness was a feature extracted from the original image, shape group and the feature name was *Flatness*.

All radiomics feature values were standardize through Z-score and presented as median (upper quantile to lower quantile). The corresponding coefficient of each feature was generated by the least absolute shrinkage and selection operator algorithm.

**P* values came from comparisons of the radiomics feature value difference between NMIBC and NIBC in each data cohort by Mann-Whitney U test.

**Supplement figures**


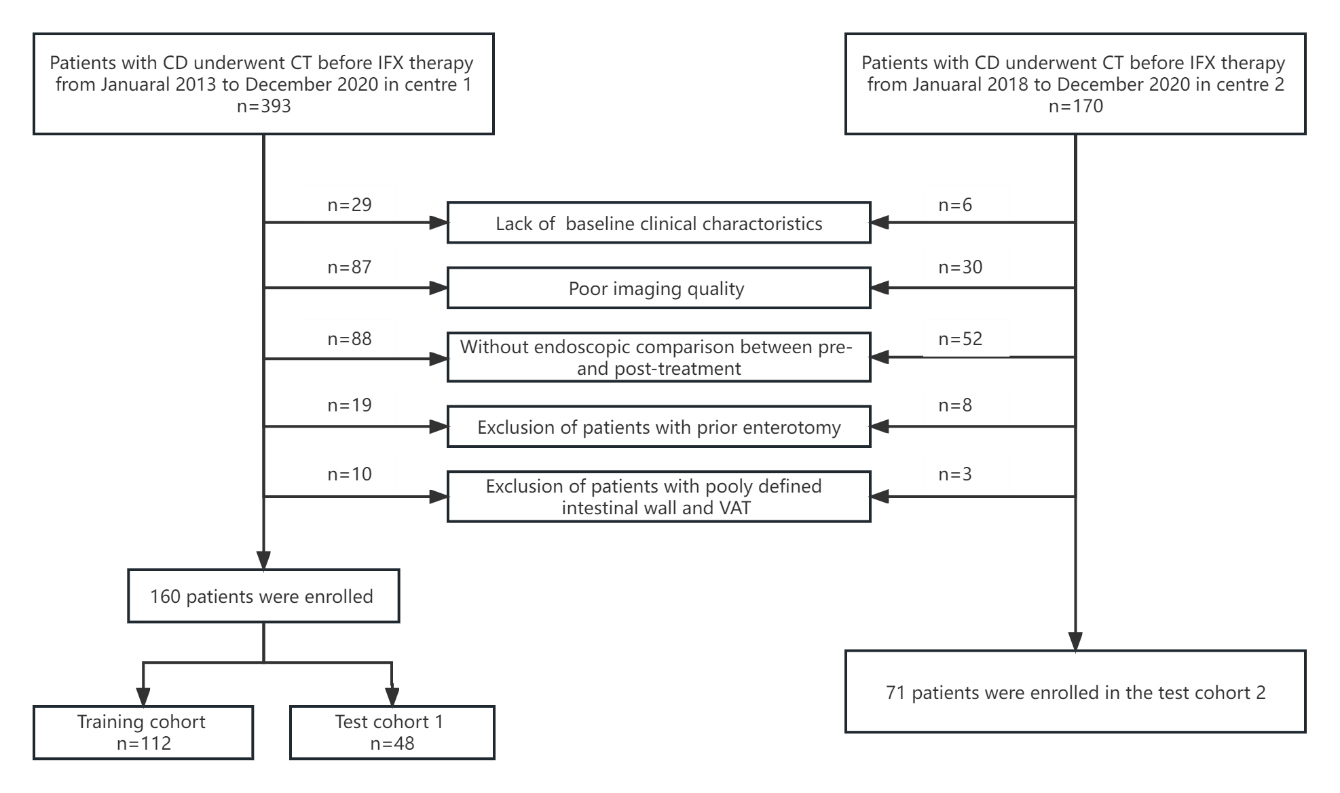


**Supplement figure 1**. Inclusion and exclusion criteria and recruitment pathways for patients in this study. (CD, Crohn's disease; IFX, infliximab; PR, primary response; PNR, primary nonresponse; Centre 1, The First Affiliated Hospital of Sun Y at-Sen University; Centre 2, The Sixth Affiliated Hospital of Sun Yat-Sen University.)

**
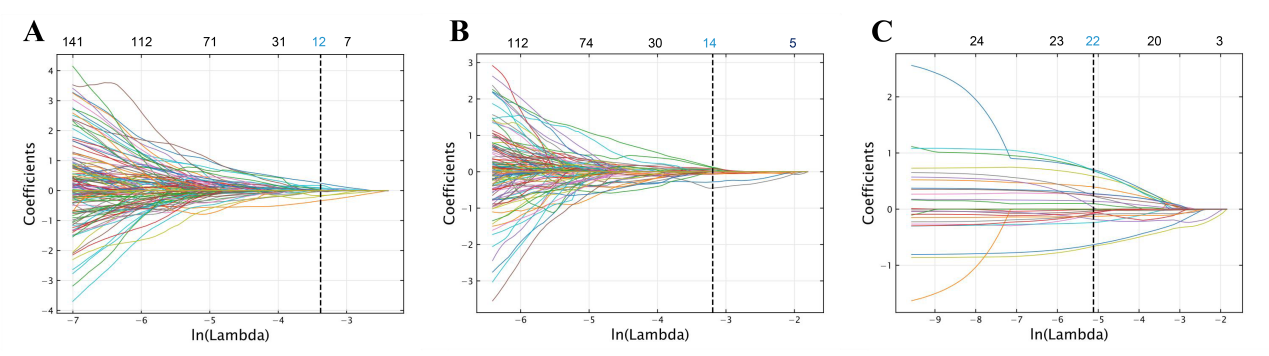
**

**Supplementary figure 2.** LASSO coefficient profile plots of the selected radiomics features in (A) VAT model, (B) bowel model and (C) VAT-bowel model. In each plot, the x-axis at the bottom represents $\ln(lambda)$, while the x-axis at the top is the number of the rest radiomics features that vary with lambda. The vertical dashed line indicates the optimal lambda value [$\ln(lambda)$= -3.381, -3.194 and -5.116, respectively], resulting in 12, 14 and 22 radiomics features with non-zero coefficients for each model finally.

(LASSO, least absolute shrinkage and selection operator; VAT model, radiomics model based on features extracted from visceral adipose tissue; bowel model, radiomics model based on features extracted from the whole inflamed bowel; VAT-bowel model, a combination of the VAT model and bowel model).

1. Xuehua Li YZ, Chenglang Yuan, Jinjiang Lin, Xiaodi Shen, Minyi Guo, Baolan Lu, Jixin Meng, Yangdi Wang, Naiwen Zhang, Zixin Luo, Guimeng Hu, Ren Mao, Minhu Chen, Canhui Sun, Ziping Li, Qing-hua Cao, Baili Chen, Zhihui Chen, Bingsheng Huang, Shi-Ting Feng. Identifying patients with Crohn's disease at high risk of primary nonresponse to infliximab using a radiomic-clinical model. International Journal of Intelligent Systems 2022, 37(12)**:** 11853-11870.
